# Effect of *Magnolia officinalis* and *Phellodendron amurense* (Relora^®^) on cortisol and psychological mood state in moderately stressed subjects

**DOI:** 10.1186/1550-2783-10-37

**Published:** 2013-08-07

**Authors:** Shawn M Talbott, Julie A Talbott, Mike Pugh

**Affiliations:** 1 SupplementWatch, 648 Rocky Knoll, Draper, UT 84020, USA; 2 MonaVie, 10855 S River Front Parkway, South Jordan, UT 84095, USA

**Keywords:** Stress, Cortisol, Vigor, Magnolia, Phellodendron, Mood, Relora

## Abstract

**Background:**

Magnolia (*Magnolia officinalis*) and Phellodendron (*Phellodendron amurense*) barks are medicinal plants commonly used as traditional remedies for reducing stress and anxiety. Modern dietary supplements are intended to induce relaxation and reduce stress as well as stress-related eating. Previous studies have shown the combination of Magnolia/Phellodendron (MP) to reduce both cortisol exposure and the perception of stress/anxiety, while improving weight loss in subjects with stress-related eating. Competitive athletes are “stressed” by their intense exercise regimens in addition to their normal activities of daily living and thus may benefit from a natural therapy intended to modulate baseline perceptions of stress and stress hormone exposure.

**Methods:**

We assessed salivary cortisol exposure and psychological mood state in 56 subjects (35 men and 21 women) screened for moderate stress and supplemented with a standardized/patented MP combination (Relora**®**, Next Pharmaceuticals) or Placebo for 4 weeks.

**Results:**

After 4 weeks of supplementation, salivary cortisol exposure was significantly (p<0.05) lower (−18%) in the Relora group compared to Placebo. Compared to Placebo, the Relora group had significantly better (p<0.05) mood state parameters, including lower indices of Overall Stress (−11%), Tension (−13%), Depression (−20%), Anger (−42%), Fatigue (−31%), and Confusion (−27%), and higher indices of Global Mood State (+11%) and Vigor (+18%).

**Conclusion:**

These results indicate that daily supplementation with a combination of Magnolia bark extract and Phellodendron bark extract (Relora**®**) reduces cortisol exposure and perceived daily stress, while improving a variety of mood state parameters, including lower fatigue and higher vigor. These results suggest an effective natural approach to modulating the detrimental health effects of chronic stress in moderately stressed adults. Future studies should examine the possible performance and recovery benefits of Relora supplementation in athletes overstressed by the physical and psychological demands of training and competition.

## Background

The relationship between chronic psychological stress and reduced health is well established [1], with psychological stress having been shown to increase susceptibility to a wide range of diseases including anxiety, depression, diabetes, and obesity [2-4]. Even the “stress” of short-term sleep loss has significant implications for long-term health and well-being due to adverse systemic health effects including suppressed immune function, abdominal obesity, insomnia, depression, and generalized fatigue [5,6].

Interventions for stress and anxiety range from nutritional support to the use of antidepressant medications such as benzodiazepines and selective serotonin reuptake inhibitors [7,8]. A United States Patent (No. 6,582,735) has been granted for the use of an extract of *Magnolia officinalis* bark for stress related conditions involving elevated cortisol, such as control of body weight, sleep disturbances and restlessness [9].

Extracts of *Magnolia officinalis* bark and its active constituent, honokiol, have been studied in animal models with comparable anxiolytic activity to diazepam (a benzodiazepine anxiolytic used to treat anxiety), but without associated side effects such as sedation [10-13]. Berberine, a constituent of the *Phellodendron* extract, has also demonstrated a significant anxiolytic effect in rodent stress studies, including the elevated plus maze test and the forced swim test [14,15]. The combination of magnolia plus phellodendron appears to be even more effective in controlling stress/anxiety compared to either herb used separately [16-19].

The subject of this study, Relora® (Next Pharmaceuticals, Inc, Salinas, CA), is a proprietary dietary supplement formulation consisting of a blend of extracts of *Magnolia officinalis* bark and *Phellodendron amurense* bark standardized to honokiol and berberine, respectively. In previous studies, Relora has demonstrated efficacy for reducing stress and anxiety in animals [18,19] and enhancing feelings of well-being in human subjects [20,21]. One study also measured the effects of Relora on salivary cortisol, finding benefits in reducing cortisol and increasing dehydroandrostenedione (DHEA) levels in stressed subjects [20].

In this study, we report the effects of using the Relora combination of magnolia bark and phellodendron bark on salivary cortisol and psychological well-being of healthy subjects under moderate levels of perceived psychological stress. The current study employed a well-validated psychological assessment known as the Profile of Mood States (POMS) to assess mood state. A key objective of the study was to explore how 4 weeks of magnolia/phellodendron supplementation (Relora versus a placebo) affected cortisol, various moods, and overall stress levels under conditions of moderate psychological stress.

## Methods

### Dietary supplement

Relora® is a proprietary blend of a patented extract of the bark of *Magnolia officinalis* and an extract of the bark of *Phellodendron amurense* (US Patent Nos. 6,582,735 and 6,814,987). The product is standardized to “not less than 1.5% honokiol and 0.1% berberine.” Subjects ingested 500 mg/day at breakfast (250 mg) and dinner (250 mg) in white opaque capsules or a look-alike placebo that was identical in size, shape and color.

### Study design

This study was done in accordance with the Helsinki Declaration, as revised in 1983, for clinical research involving humans and all procedures, measurements, and informed consent processes were reviewed and approved by an external third-party review board (Aspire IRB; Santee, CA).

Subjects signed informed consent documents after the study details were explained. The study used a randomized placebo-controlled, double-blind design. Subjects were randomly assigned, through a random number generator, to either 500 mg/day containing supplement (250 mg of Relora®, consumed at breakfast and dinner) or a look-alike Placebo (250 mg of rice flour); bottles were labeled only with a pre-assigned random code. Subjects self-administered the allotted capsule twice daily in the morning with breakfast and in the evening with dinner for 4 weeks. Subjects were contacted weekly to remind them to take their capsules daily. Empty bottles were returned after the study for a count of any unused capsules (an indicator of missed doses). Compliance with these instructions was high (data not shown).

We screened 60 subjects for moderate levels of psychological stress, with 56 subjects completing the study. Sixty (60) subjects were randomized to receive Supplement (30 subjects) or look-alike Placebo (30 subjects) for 4 weeks. The 4-week duration was selected as more representative of persistent changes in mood state that may result from superior hormone balance, as opposed to short-term changes in emotions that may be more closely linked with stressors of daily living.

At Baseline (week 0) and Post-supplementation (week 4), we assessed body weight and body fat percentage (Tanita BDF-300A bioelectrical impedance analyzer), overall stress (Yale Stress Survey), psychological mood state (Profile of Mood States Survey) and salivary cortisol. Mood State (Vigor, Depression, Anger, Confusion, Fatigue, and Anxiety) was assessed using the validated Profile of Mood States (POMS) survey [22,23]. Cortisol exposure was assessed in pooled saliva samples collected at three time points during each collection day (morning, afternoon, and evening). The morning sample was collected upon waking at approximately 6am; the afternoon sample at approximately 2pm; and the evening sample immediately before bed at approximately 10pm to represent as much of a total daily “cortisol exposure” for each subject as possible. Cortisol circadian rhythm data will be reported elsewhere. Saliva samples were analyzed for free cortisol by enzyme immunoassay (EIA; Salimetrics, State College, PA, USA).

Fifty-six subjects (35 men & 21 women, age 28±11 years) completed the study, with two women in each group lost to follow up (did not return final surveys or saliva samples).

### Mood assessment

We employed the Profile Of Mood States (POMS) questionnaire, to measure 6 primary psychological factors (tension, depression, anger, fatigue, vigor or confusion), plus the combined “global mood state” as an indication of subjective well-being. The POMS methodology has been used in nearly 3,000 studies and its validity is well established. The POMS profile uses 65 adjective-based intensity scales scored on a 0–4 hedonic scale (0 = not at all, 4 = extremely). The 65 adjective responses are categorized into the six mood factors (tension, depression, anger, fatigue, vigor or confusion), tabulated, scored and analyzed. The output of the POMS questionnaire is an assessment of the positive and negative moods of each subject at baseline and 4-weeks.

### Data management and analysis

All questionnaires were completed at a central location and transcribed to a central database. Subjects that did not complete the questionnaires or submitted incomplete questionnaires were dropped from the study and not included in the study analysis (four subjects – two females from each group). Data was identified by subject number and examined for accuracy and completeness. Tabulated data was analyzed with JMP 8.0 (SAS Institute) using standard parametric paired t-tests and significance was assessed with a two-tailed alpha level set at 0.05.

## Results

Over the course of the 4-week supplementation period, there were no adverse events or side effects reported. There were no significant changes in body weight or body fat percentage.

**Figure 1:**
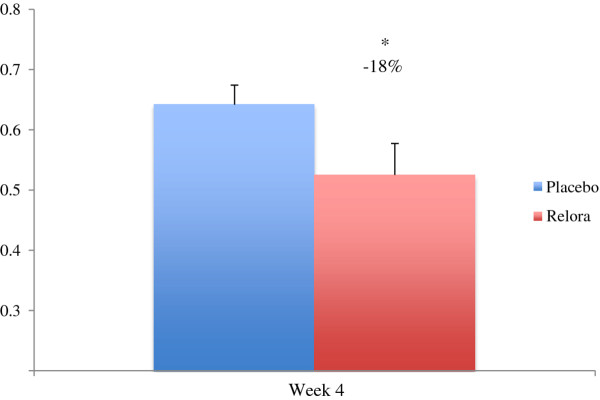
**Salivary Cortisol (ug/ml).** Salivary cortisol was 18% lower (p<0.05) in the Relora group compared to Placebo at Week 4 (0.525+0.190 to 0.642+0.353).

At week 4, salivary cortisol exposure was significantly (p<0.05) lower (−18%) in the Relora group (Figure 1).

**Figure 2:**
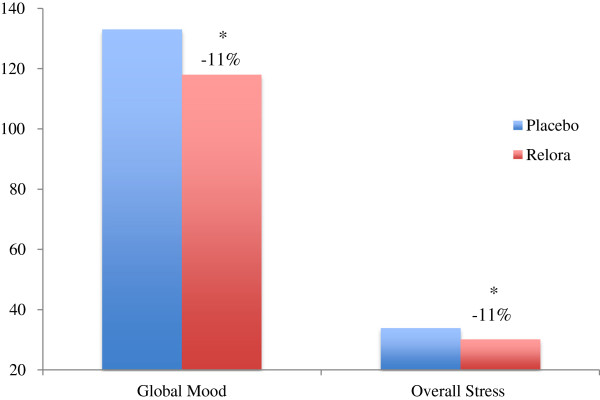
**Global Mood State (POMS) and Overall Stress (Yale Stress Survey).** Global Mood State was 11% better (p<0.05) in the Relora group compared to Placebo (118+18 to 133+30) – lower score is a “better” Global Mood State (POMS). Overall Stress (Yale Stress Survey) was 11% lower (p<0.05) in the Relora group compared to Placebo (30.2+5.2 to 33.9+7.4). The global mood state was calculated based on scoring (0-4 with 0 = not at all, 2 = moderately and 4 = extremely) answers to 58 of the 65 adjectives of the POMS (a lower number is a “better” global mood state). Global Mood State is the combined score of the 6 subscales of the POMS (McNair et al., [9]).

**Figure 3:**
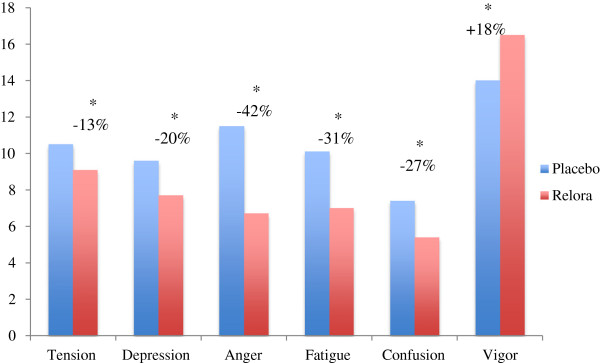
**Profile of Mood States (POMS).** Numerical scores for each of the 6 subscales of the POMS (McNair et al., [9]). The Relora group showed significantly improved mood state parameters compared to Placebo at Week 4 (* = p<0,05).

Significantly better (p<0.05) mood state indices were observed in the Relora group for Overall Stress (−11%) and Global Mood State (−11%) compared to Placebo (Figure 2). Mood State subscales (Figure 3) were significantly better (p<0.05) in the Relora group compared to Placebo at week 4; Tension (−13%), Depression (−20%), Anger (−42%), Fatigue (−31%), Confusion (−27%), and Vigor (+18%).

## Discussion

Antidepressant drugs are the most commonly prescribed class of medications in the United States and are used by athletes and non-athletes alike [24]. More than 10% of the American population is taking one or more antidepressant drugs, which represents 27 million individuals taking more than 120 million prescriptions and spending over $80 billion per year. According to a recent survey [25], large numbers of Americans feel an antidepressant drug would be helpful for; dealing with day-to-day stresses (83%); making things easier in relations with family and friends (76%); and helping people feel better about themselves (68%). However, because antidepressant drugs carry the United States Food and Drug Administration’s most stringent “black box” warning (associated with serious life-threatening adverse effects), there is need for safe and effective natural approaches to reducing stress and anxiety.

In addition to a balanced diet, regular physical activity, and various stress management techniques, certain dietary supplements may be effective in naturally maintaining the normal balance between stress, cortisol, and emotional well-being. For example, there are numerous commercial examples of general-purpose “relaxation” and “calming” teas based on traditional herbal blends such as chamomile, fennel, lemon balm and others, while magnolia and phellodendron bark extracts have been specifically demonstrated as natural anxiolytic agents, [7-21,26]. As such, appropriate dietary supplements may be a safe and effective natural adjunct to diet/exercise/stress management techniques to bring stress response and cortisol levels back to within normal ranges in individuals suffering from chronic stress or in athletes suffering from overtraining syndrome.

Magnolia bark *(Magnolia officinalis)* and Phellodendron bark (*Phellodendron amurense*) are traditional herbal medicines used since 100A.D. for treating “stagnation of Qi” in Chinese medicine [7,8,17], which is analogous to what we view in Western medicine as reduced psychological vigor or burnout. Magnolia bark extracts are rich in the phenolic compound, honokiol [12], while Phellodendron bark extracts are rich in berberine [14,15] – each of which contributes to the primary anti-stress, anti-anxiety, and cortisol-lowering effects of the plants [9-19,26]. Research has shown magnolia and phellodendron extracts and their primary bioactives (honokiol and berberine) to possess powerful “mental acuity” benefits [10,11,16] via their actions in modulating the activity of various neurotransmitters and related enzymes in the brain, including brain-derived neurotrophic factor, acetylcholine, choline acetyltransferase, and acetylcholinesterase.

Numerous animal studies have demonstrated that honokiol and berberine act as anxiolytic agents [9-19,26]. When compared to pharmaceutical agents such as Valium (diazepam), honokiol and berberine appear to be as effective in their anti-anxiety activity yet not nearly as powerful in their sedative ability [9,12,13]. These results have been demonstrated in numerous animal studies and suggest that Relora, which is standardized to both honokiol (from magnolia bark) and berberine (from phellodendron), is an effective natural approach for controlling the detrimental effects of everyday stressors, without the tranquilizing side effects of pharmaceutical agents [14-19,26]. Previous human studies on Relora have shown similar anti-stress and anxiolytic benefits in moderately stressed subjects [20,21]. The results reported in this study expand on previous findings of simple “relaxation” benefits of Relora to demonstrate specific effects on psychological mood state parameters in moderately-stressed subjects, including benefits for Global Mood State (analogous to an assessment of overall well-being), Tension, Depression, Fatigue, and Vigor (the opposite psychological state from “Burnout”). The magnitude of benefit in stress hormone (cortisol) reduction (18%) and mood state improvement (11%-42%) is meaningful from the perspective of optimal mental and physical performance. For example, the 18% higher Vigor or the 20% lower Depression score observed in the Relora group, could reasonably be associated with subjects reporting “feeling good” (in the case on our moderately-stressed subjects) or “performing well” (in the case of over-stressed or over-trained athletes, which should be the subject of future studies).

Although our study was not conducted in competitive athletes, a number of our moderately stressed healthy subjects were recreational runners and cyclists who commented about feeling more “balanced” in their workouts when their stress levels were balanced. This is a logical individual perception based on a number of studies in elite-level and recreational athletes that have found a direct relationship between overall stress (physical training and psychological stress) and athletic performance, including both mental and physical performance parameters [27-31]. Competitive athletes tend to be characterized by an elevated Vigor score and lower Fatigue score compared to non-athletes [27]. However, in many intervention studies of athletes, a dose–response exists between training stress and mood state [28,29], so as overall physical “training stress” is elevated beyond a certain tipping point, psychological mood state becomes depressed. In addition, low Vigor scores and overall reduced psychological mood state have been identified as predictors of future athletic injury [30]. The most dramatic changes in psychological mood state are logically the result of intensified periods of training (e.g. increased training intensity and/or duration), which can be modulated positively or negatively by psychological stress (e.g. exams), competitive anxiety, social support network, sleep patterns, and recovery methods [27-31]. Based on the magnitude of the positive changes in cortisol levels and mood state parameters, we would recommend further athlete-specific studies to gauge the possible mental/physical performance benefits of Relora in enhancing post-exercise recovery and preventing over-training syndrome in competitive athletes.

Results from the current study indicate that daily supplementation with a combination of magnolia bark and phellodendron bark (Relora) reduces cortisol exposure and perceived stress, while improving a variety of mood state parameters. Compared to the Placebo group, salivary cortisol exposure was significantly lower (−18%) in the Relora group, while mood state parameters were significantly and meaningfully higher, including an 11% superior Global Mood State and 18% higher Vigor, with 13% lower Tension and 20% lower Depression indices. These results indicate that daily supplementation with a combination of Magnolia and Phellodendron (Relora) is an effective natural approach to the detrimental health effects of chronic stress.

## Conclusions

The present study indicates a significant “anti-stress” benefit of magnolia/phellodendron bark (Relora) supplementation in moderately stressed non-athletes, and suggests a possible benefit for athletes to recover from “training stress” induced by the physical and psychological demands of competition and training. Future studies should examine the potential benefits of Relora in helping athletes to enhance post-exercise recovery and possibly to help prevent overtraining syndrome.

## Competing interests

This study was funded by the manufacturer of Relora (Next Pharmaceuticals) and conducted by SupplementWatch. The authors of this paper have no direct financial relationship with Next Pharmaceuticals or with the Relora dietary supplement. ST and JT are employees of SupplementWatch. ST and MP are employees of MonaVie, which markets a dietary supplement containing Relora as one of several ingredients.

## Authors’ contributions

Each author contributed significantly to the successful carriage of this study. ST designed the study and drafted the manuscript. JT coordinated the IRB approval, subject visits, and sample inventory. MP participated in the study design and coordination of subject visits. All authors read and approved the manuscript.

## References

[B1] Cohen S Janicki-Deverts D Miller GE Psychological stress and disease JAMA 2007 14 Oct 10;298:1685–7, 2007 10.1001/jama.298.14.1685 17925521

[B2] Dallman MF la Fleur SE Pecoraro NC Gomez F Houshyar H Akana SF Minireview: glucocorticoids – food intake, abdominal obesity, and wealthy nations in 2004 Endocrinology 2004 145 2633 2638 10.1210/en.2004-0037 15044359

[B3] Epel E Lapidus R McEwen B Brownell K Stress may add bite to appetite in women: a laboratory study of stress-induced cortisol and eating behavior Psychoneuroendocrinology 2001 26 37 49 10.1016/S0306-4530(00)00035-4 11070333

[B4] Epel ES McEwen B Seeman T Matthews K Castellazzo G Brownell KD Bell J Ickovics JR Stress and body shape: stress-induced cortisol secretion is consistently greater among women with central fat Psychosom Med 2000 62 623 632 11020091 10.1097/00006842-200009000-00005

[B5] Szelenberger W Soldatos C Sleep disorders in psychiatric practice World Psychiatry 2005 4 186 90 16633547 PMC1414775

[B6] Taheri S Lin L Austin D Young T Mignot E Short sleep duration is associated with reduced leptin, elevated ghrelin, and increased body mass index PloS Med 2004 1 e62 10.1371/journal.pmed.0010062 15602591 PMC535701

[B7] Weeks BS Formulations of dietary supplements and herbal extracts for relaxation and anxiolytic action: Relarian Med Sci Monit 2009 15 11 RA256 62 19865069

[B8] Lee YJ Lee YM Lee CK Jung JK Han SB Hong JT Therapeutic applications of compounds in the Magnolia family Pharmacol Ther 2011 130 2 157 76 10.1016/j.pharmthera.2011.01.010 21277893

[B9] Xu Q Yi LT Pan Y Wang X Li YC Li JM Wang CP Kong LD Antidepressant-like effects of the mixture of honokiol and magnolol from the barks of Magnolia officinalis in stressed rodents Prog Neuropsychopharmacol Biol Psychiatry 2008 32 3 715 25 10.1016/j.pnpbp.2007.11.020 18093712

[B10] Chiang J Shen YC Wang YH Hou YC Chen CC Liao JF Yu MC Juan CW Liou KT Honokiol protects rats against eccentric exercise-induced skeletal muscle damage by inhibiting NF-kappaB induced oxidative stress and inflammation Eur J Pharmacol 2009 610 1–3 119 27 19303869 10.1016/j.ejphar.2009.03.035

[B11] Harada S Kishimoto M Kobayashi M Nakamoto K Fujita-Hamabe W Chen HH Chan MH Tokuyama S Honokiol suppresses the development of post-ischemic glucose intolerance and neuronal damage in mice J Nat Med 2012 66 4 591 9 10.1007/s11418-011-0623-x 22261858

[B12] Kuribara H Stavinoha WB Maruyama Y Behavioural pharmacological characteristics of honokiol, an anxiolytic agent present in extracts of Magnolia bark, evaluated by an elevated plus-maze test in mice J Pharm Pharmacol 1998 50 819 26 10.1111/j.2042-7158.1998.tb07146.x 9720634

[B13] Kuribara H Stavinoha WB Maruyama Y Honokiol, a putative anxiolytic agent extracted from magnolia bark, has no diazepam-like side-effects in mice J Pharm Pharmacol 1999 51 97 103 10197425

[B14] Peng WH Lo KL Lee YH Hung TH Lin YC Berberine produces antidepressant-like effects in the forced swim test and in the tail suspension test in mice Life Sci 2007 81 11 933 8 10.1016/j.lfs.2007.08.003 17804020

[B15] Peng WH Wu CR Chen CS Chen CF Leu ZC Hsieh MT Anxiolytic effect of berberine on exploratory activity of the mouse in two experimental anxiety models: interaction with drugs acting at 5-HT receptors Life Sci 2004 75 20 2451 62 10.1016/j.lfs.2004.04.032 15350820

[B16] Li LF Lu J Li XM Xu CL Deng JM Qu R Ma SP Antidepressant-like effect of magnolol on BDNF up-regulation and serotonergic system activity in unpredictable chronic mild stress treated rats Phytother Res 2012 26 8 1189 94 10.1002/ptr.3706 22223265

[B17] Maruyama Y Kuribara H Morita M Yuzurihara M Weintraub ST Identification of magnolol and honokiol as anxiolytic agents in extracts of saiboku-to, an oriental herbal medicine J Nat Prod 1998 61 135 8 10.1021/np9702446 9461663

[B18] Sufka KJ Roach JT Chambliss WG Jr Broom SL Feltenstein MW Wyandt CM Zeng L Anxiolytic properties of botanical extracts in the chick social separation-stress procedure Psychopharmacology (Berl) 2001 153 2 219 24 10.1007/s002130000571 11205422

[B19] Qiang LQ Wang CP Wang FM Pan Y Yi LT Zhang X Kong LD Combined administration of the mixture of honokiol and magnolol and ginger oil evokes antidepressant-like synergism in rats Arch Pharm Res 2009 32 9 1281 92 10.1007/s12272-009-1914-6 19784585

[B20] Garrison R Chambliss WG Effect of a proprietary Magnolia and Phellodendron extract on weight management: a pilot, double-blind, placebo-controlled clinical trial Altern Ther Health Med 2006 12 1 50 54 16454147

[B21] Kalman DS Feldman S Feldman R Schwartz HI Krieger DR Garrison R Effect of a proprietary Magnolia and Phellodendron extract on stress levels in healthy women: a pilot, double-blind, placebo-controlled clinical trial Nutr J 2008 7 11 10.1186/1475-2891-7-11 18426577 PMC2359758

[B22] McNair DM Lorr M Droppleman LF Manual for the Profile of Mood States 1971 San Diego, CA: Educational and Industrial Testing Services

[B23] Leunes A Updated bibliography on the profile of mood states in sport and exercise psychology research J Appl Sport Psychol 2000 12 1 110 113 10.1080/10413200008404216

[B24] Olfson M Marcus SC National patterns in antidepressant medication treatment Arch Gen Psychiatry 2009 66 8 848 56 10.1001/archgenpsychiatry.2009.81 19652124

[B25] Harman JS Edlund MJ Fortney JC Trends in antidepressant utilization from 2001 to 2004 Psychiatr Serv 2009 60 5 611 6 10.1176/appi.ps.60.5.611 19411347

[B26] Li JM Kong LD Wang YM Cheng CH Zhang WY Tan WZ Behavioral and biochemical studies on chronic mild stress models in rats treated with a Chinese traditional prescription Banxia-houpu decoction Life Sci 2003 74 1 55 73 10.1016/j.lfs.2003.06.030 14575813

[B27] Berglund B Safstrom H Psychological monitoring and modulation of training load of world-class canoeists Med Sci Sports Exer 1994 26 8 1036 1040 7968421

[B28] Santhiago V Da Silva AS Papoti M Gobatto CA Effects of 14-week swimming training program on the psychological, hormonal, and physiological parameters of elite women athletes J Strength Cond Res 2011 25 3 825 32 10.1519/JSC.0b013e3181c69996 20571443

[B29] Pierce EF Jr Relationship between training volume and mood states in competitive swimmers during a 24-week season Percept Mot Skills 2002 94 3 Pt 1 1009 12 12081260 10.2466/pms.2002.94.3.1009

[B30] Lavallée L Flint F The relationship of stress, competitive anxiety, mood state, and social support to athletic injury J Athl Train 1996 31 4 296 9 16558413 PMC1318911

[B31] Faude O Meyer T Urhausen A Kindermann W Recovery training in cyclists: ergometric, hormonal and psychometric findings Scand J Med Sci Sports 2009 19 3 433 41 10.1111/j.1600-0838.2008.00795.x 18435693

